# Another Experience

**Published:** 1869-09

**Authors:** 


### ANOTHER EXPERIENCE.

About nine years ago we gave an account of an operation under the hands of Dr. Atkinson, in the spring of 1860, upon our second left superior molar, decayed largely upon the posterior proximal surface, involving the masticating surface to some extent, also the pulp chamber—the pulp being dead at that time, the roots and pulp chamber were filled. Upon examination, on the 10th inst, August, 1869. Some imperfection was found along the borders of the lateral walls, and slight at the cervical border. In order that the operation might be most thorough, it was deemed best to remove the filling at least from the decayed and pulp cavities, which being done, the walls for the most part were found to be in a good condition, except some odor, occasioned by leakage through the imperfect border. This was corrected, and the cavity properly excavated and prepared, and then filled with (to us the novel feature of the operation) No. 20 gold foil, and this packed with a 6 ounce lead mallet, and all this with less unpleasant sensation than had been experienced nine years ago in filling the same cavity with Nos. 4 and 6 gold foil, condensed with a wood mallet of about 3 ounces. The introduction of the former filling occupied four hours and twenty minutes; the introduction of this one about two hours. Ninety grains of gold was used in this filling, and about seventy in the former.

The cavity was slightly larger now than before, and the fissure opened across the crown to the anterior proximal surface, and then communicating with a small cavity in that surface. This was additional filling over the former one, and all this done within the two hours.

There are two or three points in connection with this case worthy of note. At the time of the first operation the pulp had been dead for some time, and an abscess was formed upon the point of the palatel root, which was destroyed at the time of the filling, as detailed in the former description. The tooth has remained free from irritation, and maintained good health, and been in active service ever since, notwithstanding its hasty destruction was foretold by many *prophets.* Another point is the manner of its recent filling; with No. 20 foil, with a lead mallet, with less annoyance than before; and in less than half the time, and considerably more work being performed.

For a few hours there was a slight periostial disturbance, but it soon passed away, and now, at every roll-call it answers " here, and ready for duty." And now we are ready for another batch of prophesies. So come on ye prophets.

Dr. ATKINSON is using No. 20 foil quite extensively and with great satisfaction. We are using these heavy foils, and shall have something to say upon the subject by and by. T.

